# A Longitudinal Study of Cognition, Proton MR Spectroscopy and Synaptic and Neuronal Pathology in Aging Wild-type and AβPPswe-PS1dE9 Mice

**DOI:** 10.1371/journal.pone.0063643

**Published:** 2013-05-22

**Authors:** Diane Jansen, Valerio Zerbi, Carola I. F. Janssen, Pieter J. W. C. Dederen, Martina P. C. Mutsaers, Anne Hafkemeijer, Anna-Lena Janssen, Cindy L. M. Nobelen, Andor Veltien, Jack J. Asten, Arend Heerschap, Amanda J. Kiliaan

**Affiliations:** 1 Department of Anatomy, Radboud University Nijmegen Medical Centre, Donders Institute for Brain, Cognition and Behaviour, Donders Centre for Neuroscience, Nijmegen, The Netherlands; 2 Department of Radiology, Radboud University Nijmegen Medical Centre, Nijmegen, The Netherlands; Max Planck Institute of Psychiatry, Germany

## Abstract

Proton magnetic resonance spectroscopy (^1^H MRS) is a valuable tool in Alzheimer’s disease research, investigating the functional integrity of the brain. The present longitudinal study set out to characterize the neurochemical profile of the hippocampus, measured by single voxel ^1^H MRS at 7 Tesla, in the brains of AβPPSswe-PS1dE9 and wild-type mice at 8 and 12 months of age. Furthermore, we wanted to determine whether alterations in hippocampal metabolite levels coincided with behavioral changes, cognitive decline and neuropathological features, to gain a better understanding of the underlying neurodegenerative processes. Moreover, correlation analyses were performed in the 12-month-old AβPP-PS1 animals with the hippocampal amyloid-β deposition, TBS-T soluble Aβ levels and high-molecular weight Aβ aggregate levels to gain a better understanding of the possible involvement of Aβ in neurochemical and behavioral changes, cognitive decline and neuropathological features in AβPP-PS1 transgenic mice. Our results show that at 8 months of age AβPPswe-PS1dE9 mice display behavioral and cognitive changes compared to age-matched wild-type mice, as determined in the open field and the (reverse) Morris water maze. However, there were no variations in hippocampal metabolite levels at this age. AβPP-PS1 mice at 12 months of age display more severe behavioral and cognitive impairment, which coincided with alterations in hippocampal metabolite levels that suggest reduced neuronal integrity. Furthermore, correlation analyses suggest a possible role of Aβ in inflammatory processes, synaptic dysfunction and impaired neurogenesis.

## Introduction

The cause of Alzheimer’s disease (AD) is still largely unknown despite many years of extensive research. Since AD is characterized by the presence of neurofibrillary tangles and amyloid-β (Aβ) containing aggregates, it has been suggested that the Aβ peptide is a major contributor to the neurodegenerative processes in AD [1], [2]. The Aβ peptide is derived through the proteolytic cleavage of the amyloid-β precursor protein (AβPP) by the β- and γ-secretases, BACE and Presenilin (PS), and it accumulates in the brain as neuritic plaques or as vascular wall deposits that may cause cerebral amyloid angiopathy (CAA) [3], [4]. In recent years, research focus has shifted from the insoluble Aβ plaques to the soluble oligomeric forms of Aβ as potential culprits of AD. Several lines of evidence have indicated that soluble oligomers of Aβ may be responsible for synaptic dysfunction and cognitive impairment in AD patients and transgenic animal models [5], [6].

Besides Aβ, several other potential causal mechanisms have been proposed since the discovery of AD. Large epidemiological studies have revealed that many risk factors for AD are vascular-related, causing chronic cerebral hypoperfusion and cerebrovascular pathology [7]–[11], suggesting that vascular disorders may play an important role in the onset of AD. Another process that has been implicated in the onset and development of AD is chronic neuroinflammation [12]–[14]. Activated astrocytes and microglia are found in abundance near Aβ plaques and neurons at risk in AD brains, producing several proinflammatory signal molecules, including cytokines, growth factors, complement molecules, and adhesion molecules [15], [16]. Furthermore, several studies have shown that activated microglia can suppress hippocampal neurogenesis [17]–[19], thereby contributing to cognitive dysfunction in aging and AD.

Rather than one sole mechanism, it is much more likely that AD is a multifactorial disease, caused by a combination of these factors, compromising the functional integrity of the brain. One method of determining the functional integrity of the brain, or specific brain regions, is to examine their metabolism by using proton magnetic resonance spectroscopy (^1^H MRS). ^1^H MRS allows the non-invasive *in vivo* analysis of certain neurometabolites that indicate biochemical changes in the brain, which are thought to be related to the pathological processes at the molecular or cellular level [20], [21]. Altered metabolic profiles, detected by ^1^H MRS, have been reported in patients with AD [22], [23]. The most consistent findings in AD patients are a reduction of the metabolite *N*-acetylaspartate (NAA) and an elevation of the metabolite *myo*-Inositol (*m*I) in several brain regions including the hippocampus [20], [24], [25]. NAA is considered to be a marker of neuronal viability, and a reduction of NAA is commonly interpreted as a result of neuronal dysfunction or neuronal loss [26]. An elevation of *m*I has been associated with inflammatory processes, since *m*I is a putative marker for microglia and astrogliosis [27]. Furthermore, disturbances of several other metabolites have been found in AD patients, although the reports are inconsistent. Some studies identified elevated choline-containing compounds (tCho) and creatine (Cre) in AD patients [28]–[30], whereas others did not [24], [31].

Similar to AD patients, the most consistent finding in transgenic animal models is a reduction of NAA, although this was found to occur at different ages in different transgenic species apparently depending on the interplay of mouse strain, transgene and disease progression [32]. Furthermore, conflicting results have been reported for several other metabolite levels, including *m*I, taurine (Tau) and glutamate (Glu), even within the same transgenic animal model [33]. Since ^1^H MRS has great potential for the early diagnosis of AD, monitoring disease progression and evaluating the efficacy of potential therapeutic agents, it is important to characterize the alterations in neurometabolites in several transgenic animals models of AD.

The present longitudinal study set out to characterize the neurochemical profile of the hippocampus, measured by ^1^H MRS, in the brains of AβPPswe-PS1dE9 and wild-type mice at 8 and 12 months of age. Furthermore, we wanted to determine whether alterations in hippocampal metabolite levels coincided with behavioral changes, cognitive decline and neuropathological features, to gain a better understanding of the underlying neurodegenerative processes. Moreover, the extracellular amyloid-β plaque load, TBS-T soluble Aβ levels and high-molecular weight Aβ aggregate levels were determined in the brains of the 12-month-old AβPP-PS1 mice used in the present study [34]. We performed correlation analyses with these Aβ measures, to gain a better understanding of the possible involvement of Aβ in the neurochemical and behavioral changes, cognitive decline and neuropathological features in the AβPP-PS1 transgenic mice. Providing well characterized AD animal models and better understanding of the underlying pathological processes in AD is required for the development and evaluation of potential therapeutic targets.

## Animals, Materials and Methods

### Ethics Statement, Animals and Housing Conditions

The experiments were performed according to Dutch federal regulations for animal protection and were approved by Veterinary Authority of the Radboud University Nijmegen Medical Centre (Permit Number: RU-DEC2008-126). All efforts were made to minimize suffering of the animals.

The AβPPswe-PS1dE9 founders were obtained from Johns Hopkins University, Baltimore, MD, USA (D. Borchelt and J. Jankowsky, Dept. of Pathology) and a colony was established at the Radboud University Nijmegen Medical Centre, the Netherlands. In short, mice were created by co-injection of chimeric mouse/human AβPPswe (mouse AβPP695 harboring a human Aβ domain and mutations K595N and M596L linked to Swedish familial AD pedigrees) and human PS1dE9 (deletion of exon 9) vectors controlled by independent mouse prion protein promoter elements. The two transfected genes co-integrate and co-segregate as a single locus [35], [36]. This line (line 85) was originally maintained on a hybrid background by backcrossing to C3HeJ × C57BL6/J F1 mice (so-called pseudo F2 stage). For the present work, the breeder mice were backcrossed to C57BL6/J for 9 generations to obtain mice for the current study. Throughout the experiment animals were housed in groups of 2–3 mice per cage in a controlled environment, homogenously illuminated by normal fluorescent room light at 60 lux, with room temperature at 21°C, and an artificial 12∶12 h light:dark cycle (lights on at 7 a.m.). Food and water were available *ad libitum*.

Male transgenic AβPP-PS1 mice and their wild-type littermates underwent behavioral testing and MRI measurements at 8 months of age, and again at 12 months of age. In total 25 mice were used: at 8 months of age, 15 wild-type and 10 AβPP-PS1 mice, and at 12 months of age, 9 wild-type and 7 AβPP-PS1 mice. Due to some technical problems during the experiments, not all mice could be used for the statistical analyses for each measure. For example, some mice were excluded from further analyses of the ^1^H MRS data, since the spectra obtained did not meet the inclusion criteria. The body weights of the mice were determined one day before the start of the behavioral tests, and again on the day of the MRI measurements.

### Behavioral Analyses

Behavioral testing was performed in the following order: First open field, followed by Morris water maze (MWM), and finally the reversal MWM (rMWM). All testing sessions were performed during the light phase (between 9 a.m. and 5 p.m.) and were recorded for computer-assisted analysis using Noldus Ethovision 3.1 software (Noldus Information Technology B.V., Wageningen, the Netherlands). All behavioral testing was performed in the same room, homogenously illuminated by normal fluorescent room light at 60 lux.

#### Open field

To analyze explorative and anxiety-related behavior, mice were placed individually in the center of a square open field (50×50×50 cm) with white Plexiglas walls, and were observed for 30 minutes. The duration (seconds) of walking, wall leaning, rearing, sitting and grooming were scored and later analyzed in three blocks of 10 minutes. These open field parameters were defined as described previously [37], [38]. In addition, total walking distance, mean velocity, and the time spent in the corners respectively the center of the open field were obtained from the recorded sessions. The center of the open field was defined as a square measuring 20×20 cm, and the corners of the open field were defined as the sum of all four 10×10 cm squared corners.

#### Morris water maze (MWM)

To investigate spatial learning abilities, mice were tested in the Morris water maze (MWM). In short, mice were placed in a pool (104 cm diameter) filled with water (21–22°C; made opaque by the addition of milk powder) at different starting positions and trained to find a submerged platform by using distant visual cues in the room. These spatial cues were present on the four walls of the test room at a distance of 0.5 meter. The 8 cm diameter round platform was submerged 1 cm below the water surface and placed in the middle of the northeast (NE) quadrant at a distance of 26 cm from the wall. During all trials the researcher was present and always located at the same location in the room (close to the SW quadrant).

Acquisition (spatial learning): Mice were trained to find the location of the submerged escape platform in 4 acquisition trials (maximal swimming time 120 s; 30 s on the platform; inter-trial interval 60 min) per day during 4 consecutive days. The latency time (s) to find the hidden platform was scored. Starting positions during the 4 trails/day were: S, N, E, W. After the 2 min swim the mice were placed back in their home cage, and a paper towel was available inside the cage for additional drying.

Probe (spatial memory): All mice performed a single probe trial 60 min after the last trial on day 4, in which the platform was removed from the swimming pool. Mice were allowed to swim for 60 s and the time spent swimming and searching in the NE quadrant (where the platform had been located), total swimming distance, mean velocity and total number of platform crossings (at the former platform location) were recorded.

#### Reverse morris water maze (rMWM)

Four days after the standard MWM probe trial, a simplified reversal MWM [38] was performed in which the platform location was changed to the southwest (SW) quadrant. In this procedure, earlier platform location need to be encoded in the long-term memory. Memory retrieval needs to be selective for the most recently learned location, introducing an episodic like component in the spatial memory task [39]. Acquisition and probe sessions were performed similar to the standard MWM sessions, except that starting positions were E, W, S, and N, the target quadrant was SW, and training lasted only 2 days (4 trials/day).

### Magnetic Resonances Spectroscopy (MRS)

MR measurements were performed on a 7T/300 mm horizontal-bore MR spectrometer interfaced to a ClinScan console (Bruker Biospin, Ettlingen, Germany). An integrated circular polarized transmit ^1^H volume coil (200 mm/154 mm outer/inner diameter) combined with a circular polarized receive ^1^H surface coil was used for signal reception. During the experiments, mice were anesthetized with 2% isoflurane (Abott, Cham, Switzerland) in a mixture of N_2_O and oxygen (1∶2) through a nose cone. Mice were placed in a stereotactic holder to prevent unwanted movement during the scanning. Body temperature was maintained at a physiological level with heated airflow and was monitored with a rectal optical temperature probe. Respiration of the animal was monitored using a pneumatic cushion respiratory monitoring system (Small Animal Instruments Inc, NY, USA). Multislice turbo spin echo images in the coronal, transversal and longitudinal orientation were acquired to visualize the anatomy and the morphology of the mouse brain structures. Imaging parameters were: FOV = 25×25 mm, matrix size = 256×256, slice thickness = 0.5 mm, TE = 46 ms, and TR = 3500 ms.

**Figure 1:**
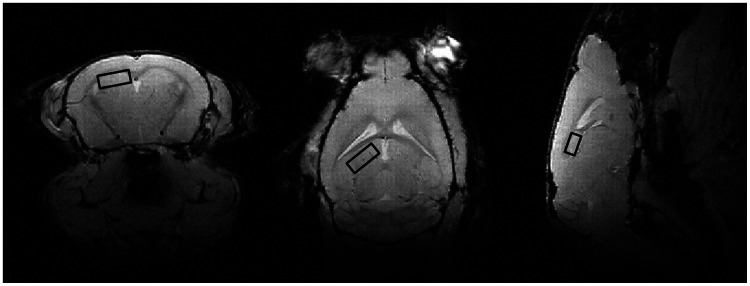
Localization of the spectroscopic volume of 1.0×1.0×1.6 mm placed in the hippocampus.

Metabolite concentrations in the hippocampus were determined using proton magnetic resonance spectroscopy (^1^H MRS) with a single voxel technique. The spectroscopic volume of interest (VOI) of 1.0×1.0×1.6 mm was positioned in the hippocampus (Figure 1), according to the mouse brain atlas of Franklin and Paxinos [40]. Water-suppressed ^1^H-MRS spectra were acquired with the stimulated echo acquisition mode (STEAM) sequence with experiment parameters: TR = 1500 ms, TE = 13 ms, and 1024 signal averages. Water suppression was performed with variable pulse power and optimized relaxation delays (VAPOR). For each ^1^H MRS spectrum, a water reference spectrum was acquired without water suppression with experiment parameters: TR = 1500 ms, TE = 13 ms, and 32 signal averages. Total acquisition time for ^1^H MRS was 27 min per animal.

Quantification of the metabolite concentration was performed using a the Linear Combination (LC) model software package (LCModel™, S. Provencher, Oakville, Canada). The quantification algorithm of LCModel™ applies linear combinations of model spectra to calculate the best fit of the experimental spectrum. The model spectra (dataset of prior knowledge) are calibrated to match the magnetic field strength, sequence type and sequence parameters used for data acquisition. The final analysis is performed in the frequency domain with raw data (free induction decay (FID)) as the input. The unsuppressed water spectrum was used to estimate the absolute concentration of the metabolites, of which simulated model spectra, generated by NMRSIM™ (Bruker Biospin, Ettlingen, Germany) were taken into the analysis.

The criteria to select reliable metabolite concentrations were based on the Cramér-Rao lower bounds (CRLB), which are estimates of the S.D. of the fit for each metabolite [41] and are also determined by LCModel™. Only fit results with a CRLB ≤20% were included in the analysis. Concentrations with CRLB>20% were classified as not accurately detectable. Based on these criteria, 2 wild-type and 2 AβPP-PS1 mice at 8 months of age, and 2 wild-type and 3 AβPP-PS1 mice at 12 months of age were excluded from further analyses, since they displayed fit results with CRLB>20%. Only metabolite fits that had a CRLB ≤20% in more than 80% of the spectra were included, and individual concentrations with corresponding CRLB>20% were not taken into account.

Seven metabolites fulfilled the criteria: choline+glycerophosphocholine+phosphocholine (tCho), creatine+phosphocreatine (tCre), glutamate (Glu), glutamine+glutamate (Glx), *myo*-Inositol+glycine (*m*I+Gly), *N*-acetylaspartate (NAA) and taurine (Tau). Although the exact function of these metabolites are not fully known, NAA is considered to be a marker of neuronal viability, tCre is involved in energy metabolism, *m*I is a putative marker for microglia and astrogliosis, and tCho is required for the synthesis of the neurotransmitter acetylcholine, and of phosphatidylcholine, a major constituent of membranes, and is therefore associated with membrane turnover [27].

### Immunohistochemistry

Directly following the MR measurements at 12 months of age, mice were sacrificed by cervical dislocation, and subsequently brains were removed from the skull after decapitation. Brains were weighed and cut mid sagittal for immunohistochemistry and biochemistry. One hemisphere was snap frozen in liquid nitrogen and then stored at −80°C, before further biochemical processing. The other hemisphere was immersion fixated in 4% paraformaldehyde for 24 hours, and subsequently stored in 0.1 M phosphate buffered saline (PBS, pH = 7.3) with 1% sodium azide at 4°C. Before cutting, the brain tissue was cryoprotected by immersion in 30% sucrose in 0.1 M phosphate buffer (PB, pH = 7.3). Six series of 40 µm coronal sections were cut through the brain using a sliding microtome (Microm HM 440 E, Walldorf, Germany). For every staining, one complete series with 240 µm distance between the sections was used. Immunohistochemistry was performed using standard free-floating labeling procedures [42], and was carried out on a shaker table at room temperature.

Presynaptic boutons were visualized with anti-synaptophysin antibody (1∶20,000; monoclonal rabbit anti-synaptophysin clone EP1098Y, Abcam Inc., Cambridge, UK) using one subseries of brain sections per animal. Synaptophysin is localized in small synaptic vesicles of the presynaptic terminal and functions in the regulation of exocytosis [43]. Donkey anti-rabbit biotin 1∶1500 (Jackson ImmunoResearch, West Grove, PA, USA) was used as secondary antibody.

Immature neurons were visualized with anti-doublecortin antibody (1∶3000; polyclonal goat anti-doublecortin (C18): sc-8066, Santa Cruz Biotechnology, Inc., Santa Cruz, CA, USA) using one subseries of brain sections per animal. Doublecortin is a microtubule-associated protein that is exclusively found in somata and processes of migrating and differentiating neurons [44], [45]. Donkey-goat biotin 1∶1500 (Jackson ImmunoResearch, West Grove, PA, USA) was used as secondary antibody.

### Quantification

Quantification of presynaptic boutons and doublecortin-positive immature neurons was performed using a Zeiss Axioskop microscope, equipped with hardware and software from Microbrightfield (Williston, VT, USA). Appropriate sections were digitized and photomicrographed using a computer-assisted analysis system, Stereo Investigator (Microbrightfield). Brain regions were based on the mouse brain atlas of Franklin and Paxinos [40]. All measurements were performed double blind by two independent raters, and measurements were averaged to obtain a single value per animal for every region of interest.

#### Quantification of synaptophysin-immunoreactive presynaptic boutons

**Figure 2:**
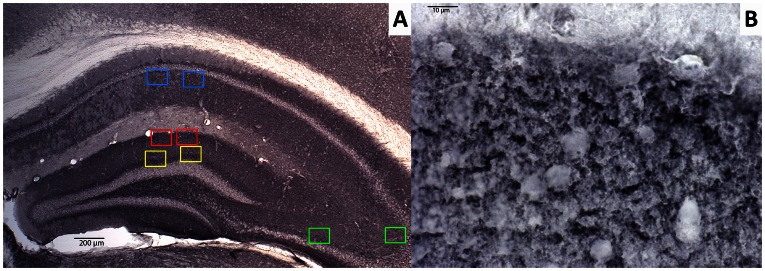
Representative image of synaptophysin-immunoreactive presynaptic boutons (SIPBs) in the hippocampus of a 12-month-old wild-type mouse. A: In the hippocampus SIPBs were analyzed in the inner (yellow) and outer (red) molecular layer of the dentate gyrus, stratum radiatum (SR) of the CA1 area (blue), and stratum lucidum (SL) of the CA3 area (green). Scale bar = 200 µm. B: SIPBs were quantified with an 100× objective using image analysis from digitized photomicrographs of the synaptophysin-immunoreactivity. Scale bar = 10 µm.

To determine the amount of synaptophysin-immunoreactive presynaptic boutons (SIPBs) in the hippocampus and cortical regions, appropriate sections were digitized and photomicrographed using an 100× oil immersion objective. SIPBs were analyzed in the hippocampal regions stratum radiatum (SR) of the cornu ammonis (CA)1 area, stratum lucidum (SL) of the CA3 area, inner molecular layer (IML) and outer molecular layer (OML) of the dentate gyrus (DG), and in the cortical regions prelimbic area (PLA) and anterior cingulate gyrus (ACg). These regions were chosen because of their large amyloid load in AD patients and transgenic mouse models for AD and their importance in learning and memory [46], [47]. The ACg was quantified at level +1.10 up to +0.86 anterior to bregma using one appropriate section per animal, the PLA was quantified at +1.98 up to +1.78 anterior to bregma using one appropriate section per animal, and hippocampal regions were quantified at −2.18 up to −2.46 posterior to bregma using one appropriate section per animal. For every region of interest, two square boxed were placed within the borders of the intended area using an 2.5 or 5× objective and images were taken using an 100× oil immersion objective (Figure 2). Images were further processed using ImageJ software (U.S. National Institutes of Health, Bethesda, MD, USA) for the quantification of the amount of SIPBs. All settings were kept identical for all analyses and background levels were equalized using a threshold. Shading correction was performed before measurement to correct for irregularities in illumination in the microscopic field. A differential contrast enhancement filter was applied to selectively enhance weak differences in contrast. To eliminate noise signal and to differentiate between possible artifacts and specific SIPBs, particles were classified based on size. Particles ranging between 0.1–4.5 µm^2^ were considered to be normal sized SIPBs [48], [49], and were included for statistical analyses. Particles smaller than 0.1 µm^2^ and larger than 4.5 µm^2^ were excluded for analyses. The amount of SIPBs/µm^2^ was defined as the number of particles divided by the total area analyzed.

#### Quantification of doublecortin-positive cells

**Figure 3:**
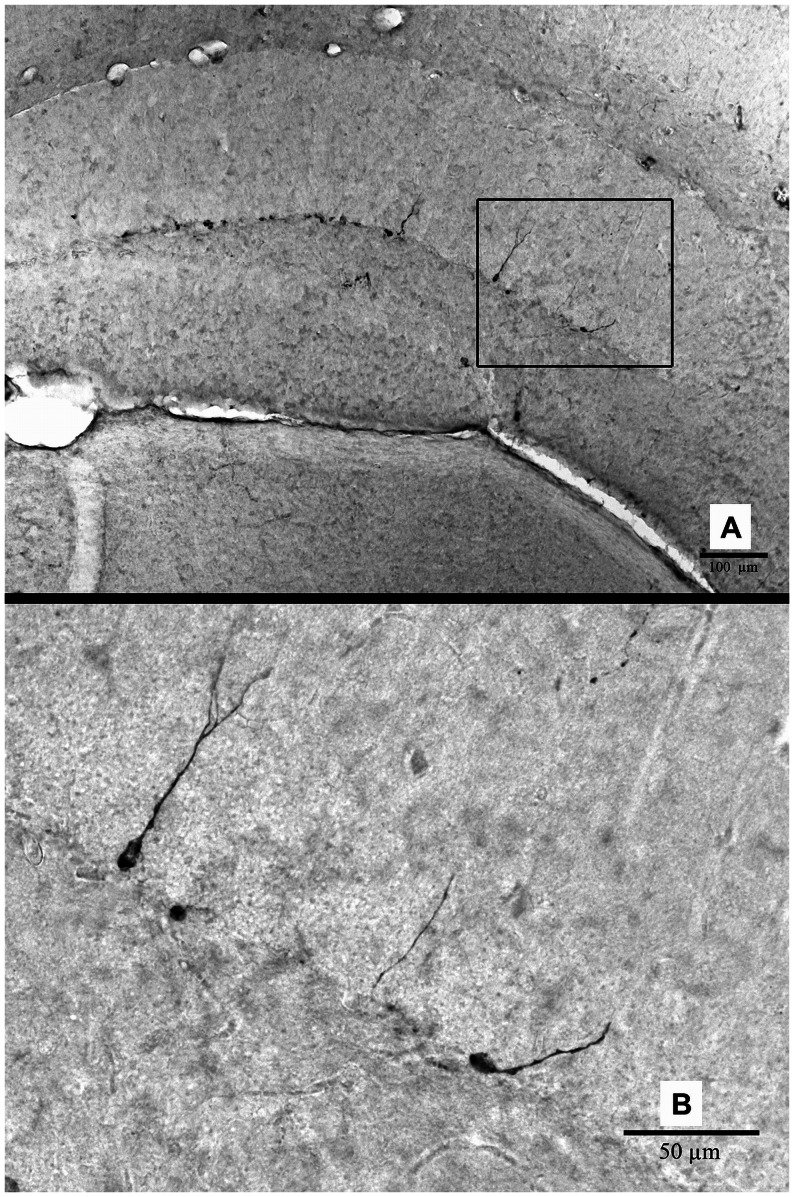
Representative image of doublecortin-positive (Dcx+) cells in the hippocampus of a 12-month-old wild-type mouse. A: Image taken using an 10× objective. Scale bar = 100 µm. B: Image taken with an 40× objective. Scale bar = 50 µm.

For the assessment of immature neurons in the hippocampus as a measure for neurogenesis (Figure 3), three alternating sections per animal (at −2.18. −2.46 and −2.70 posterior to bregma) were digitized and contours were drawn along the borders of the hippocampus using an 5× objective using Stereo Investigator software. Doublecortin-positive (Dcx+) cells were counted with an 20× objective, and the values of the three alternating sections were averaged to obtain a single value per animal.

### Amyloid-β Measures

The extracellular Aβ load, soluble Aβ levels and insoluble high-molecular weight Aβ aggregate levels were determined in the brains of the 12-month-old AβPP-PS1 mice, as has been described elsewhere [34].

**Figure 4:**
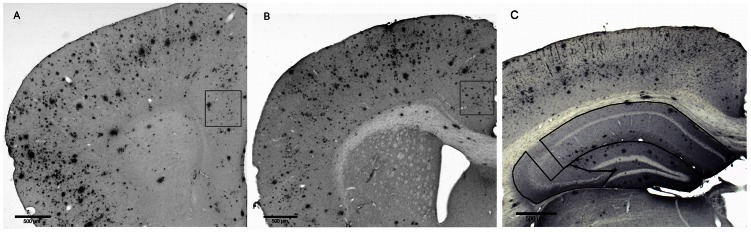
Representative images of the amyloid-β plaque load in the brain of a 12-month-old AβPP-PS1 mouse. A: The Aβ plaque load was quantified in the prelimbic area at level +1.98 up to +1.78 anterior to bregma, B: in the anterior cingulate gyrus at level +1.10 up to +0.86 anterior to bregma, and C: in the dentate gyrus (DG), CA1 and CA3 areas of the hippocampus at level −2.18 up to −2.46 posterior to bregma, using one appropriate section per animal. Images were taken using a 2.5× objective. Scale bar = 500 µm.

In short, Aβ deposits were visualized using WO-2 antibody (1∶20,000, mouse anti-human Aβ_4–10_, a kind gift of K. Beyreuther, University of Heidelberg, Germany) using one subseries of brain sections per animal. Donkey anti-mouse biotin (1∶1500, Jackson ImmunoResearch was used as secondary antibody. Extracellular Aβ plaque load was quantified in the hippocampus, prelimbic area (PLA) and anterior cingulate gyrus (ACg) with a computer-assisted analysis system (Stereo Investigator, Microbrightfield) using Cavalieri’s probe (Figure 4). The Aβ plaque load was defined as the percentage of area covered by Aβ.

For biochemical Aβ analyses, frozen hemibrains were homogenized in Tris buffered saline with 1% Triton X-100 (TBS-T) plus protease inhibitor cocktail (Roche Applied Science, Mannheim, Germany) and centrifuged at 16,000 *g* for 30 min at 4°C.The supernatant, enriched with oligomeric Aβ, was collected and stored at −80°C. The pellet, containing mainly highly aggregated Aβ, was resuspended in guanidine chloride buffer and extracted for 4 hours. The TBS-T and guanidine HCl extracts were analyzed for human Aβ_40_ and Aβ_42_ (KHB3442 and KH3482, Invitrogen, Karlsruhe, Germany) according to the manufacturers protocol. Results were normalized to the protein concentration of the sample (Bio-Rad Protein Assay, Bio-Rad Laboratories, Munich, Germany).

### Statistical Analysis

Data are expressed as mean±SEM and were analyzed with SPSS for windows 16.0 software (SPSS Inc. Chicago, IL, USA). The repeated measures ANOVA was used for the open field parameters (with the repeated measure: time) and the acquisition phase of the MWM and rMWM (with the repeated measure: trial block), followed by a Bonferroni post hoc to analyze possible interactions between time/trial block and genotype. If interactions between time/trial block and genotype (between-group-factors) were present, the data were split for the concerning factor and thereafter analyzed again with the repeated measures ANOVA. Multivariate ANOVA’s were conducted with between group factor: genotype, to analyze possible differences between wild-type and AβPP-PS1 mice in the probe trials of the MWM and rMWM, the body weight, brain weight, metabolite concentrations, and the amount of SIPBs and immature neurons. Aging effects in the AβPP-PS1 mice are represented as relative values compared to the wild-type mice (set as 100%) of the corresponding age-group, and were analyzed with between group factor: age. Correlation analyses with the Aβ measures were performed using bivariate Pearson’s correlation method. For clarity reasons, F-values are not displayed. Statistical significance was set at *p*<0.05.

## Results

### Body and Brain Weight

All mice were weighed one day before starting the behavioral test battery and again on the day of the MR measurements. Since body weights within the groups did not change significantly between those two time points, the mean weight was used for further statistical analyses. Both at 8 months and 12 months of age, AβPP-PS1 mice had a tendency towards a higher body weight than wild-type mice, but it did not reach statistical significance. At 8 months of age, overall mean body weight of the AβPP-PS1 mice was 29.79±0.65 g compared to 28.24±0.42 g of the wild-type mice (*p* = 0.051). At 12 months of age, overall mean body weight of the AβPP-PS1 mice was 33.03±1.23 g compared to 30.13±0.76 g of the wild-type mice (*p = *0.060).

Both relative and absolute brain weights were not affected by genotype (*p = *0.812 and *p* = 0.165 respectively). Overall mean brain weight of the AβPP-PS1animals was 0.49±0.01 g, which was 1.46±0.08% of their total body weight. Overall mean brain weight of the wild-type mice was 0.45±0.02 g, which was 1.49±0.07% of their total body weight.

### Behavioral Analyses

#### Open field

**Figure 5:**
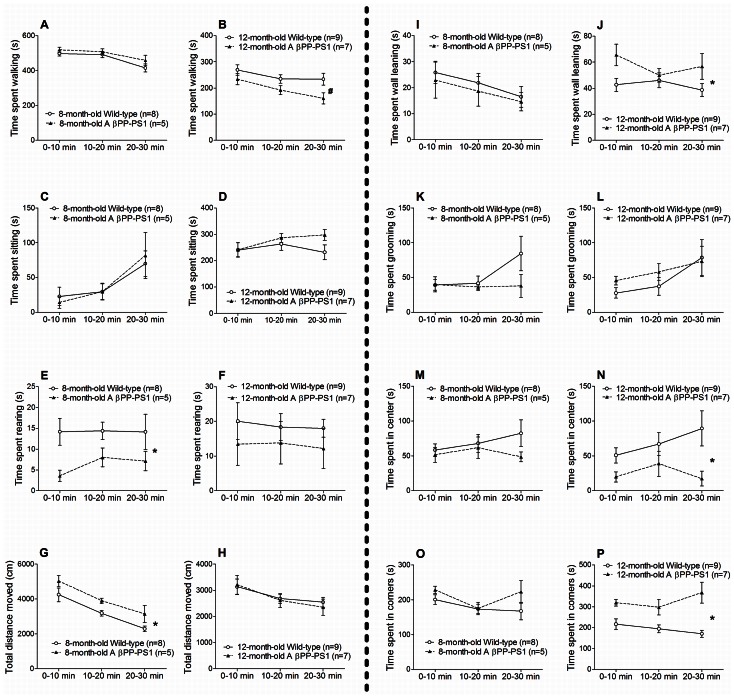
Open field behavior in AβPP-PS1 and wild-type mice at 8 and 12 months of age. Different open field parameters were measured within a 30 min period, and analyzed in three 10 min trial blocks. A and B: AβPP-PS1 mice (n = 5) did not differ from wild-type mice (n = 8) at 8 months of age (A), but spent slightly less time walking than wild-type mice at 12 months of age, # trend *p* = 0.058 (B). C and D: The duration of sitting was similar among wild-type and AβPP-PS1 mice at 8 (C) and at 12 months of age (D). E and F: AβPP-PS1 mice spent less time rearing than wild-type mice at 8 months of age, *p<0.05 (E), but did not differ from wild-type mice at 12 months of age (F). G and H: AβPP-PS1 mice traveled a longer distance than wild-type mice at 8 months of age, *p<0.05 (G), but did not differ from wild-type mice at 12 months of age (H). I and J: The duration of wall leaning was similar among wild-type and AβPP-PS1 mice at 8 months of age (I), but was increased in AβPP-PS1 mice at 12 months of age, *p<0.05 (J). K and L: Both at 8 (K) and 12 months of age (L), the time spent grooming was similar among wild-type and AβPP-PS1 mice. M and N: AβPP-PS1 mice did not differ from wild-type mice at 8 months of age (M), but spent less time in the center of the open field than wild-type mice at 12 months of age, *p<0.05 (N). O and P: AβPP-PS1 mice (n = 7) spent more time in the corners of the open field than wild-type animals (n = 9) at 12 months of age, *p<0.05 (P), but did not differ from wild-type at 8 months of age (O).

During the 30 min observation at 8 months of age, both in wild-type and AβPP-PS1 mice the time spent walking (Figure 5A; *p*<0.001) and wall leaning (Figure 5I; *p* = 0.038) decreased, as well as the distance traveled (Figure 5G; *p*<0.001) and mean velocity (*p*<0.001). Accordingly, the time spent sitting (Figure 5C; *p*<0.001) increased during the 30 min observation period. The time spent rearing (Figure 5E; *p* = 0.632) and grooming (Figure 5K; *p* = 0.263), as well as the time spent in the center (Figure 5 M; *p* = 0.673) and in the corners (Figure 5O; *p* = 0.106) of the open field remained constant over time.

8-month-old AβPP-PS1 mice were more active in the open field than wild-type mice:

AβPP-PS1 mice traveled a longer distance (Figure 5G; *p* = 0.048) and had a higher mean walking speed than wild-type mice (*p* = 0.043; wild-type 5.6±0.3 cm/s, AβPP-PS1 6.9±0.5 cm/s), although the time spent walking and sitting did not differ from the wild-type animals (Figure 5A; *p* = 0.293 and Figure 5C; *p* = 0.964 respectively). AβPP-PS1 mice also spent less time rearing than wild-type mice (Figure 5E; *p* = 0.043). No differences were observed between the genotypes in the time spent wall leaning (Figure 5I; *p* = 0.642) and grooming (Figure 5K; *p* = 0.259), and in the time spent in the center (Figure 5 M; *p* = 0.274) and corners of the open field (Figure 5O; *p* = 0.189).

Like at 8 months of age, the time spent walking (Figure 5B; *p* = 0.001), the distance traveled (Figure 5H; *p* = 0.001), and mean velocity (*p* = 0.001) decreased over time in both wild-type and AβPP-PS1 mice at 12 months of age. In contrast, the time spent grooming increased at this age during the 30 min open field (Figure 5L; *p* = 0.009), whereas the time spent sitting (Figure 5D; *p* = 0.104) and wall leaning (Figure 5J; *p* = 0.443) remained constant throughout the observation period. Again, the time spent rearing (Figure 5F; *p* = 0.866), as well as the time spent in the center (Figure 5N; *p* = 0.389) and the corners (Figure 5P; *p* = 0.465) of the open field did not change over time.

Compared to wild-type mice, 12-month-old AβPP-PS1 mice spent more time wall leaning (Figure 5J; *p* = 0.041), and slightly less time walking (Figure 5B; *p* = 0.058), although the distance traveled (Figure 5H; *p* = 0.859) and mean velocity of the AβPP-PS1 mice did not differ from the wild-type animals (*p* = 0.886; wild-type 4.7±0.3 cm/s, AβPP-PS1 4.7±0.5 cm/s). Furthermore, AβPP-PS1 mice spent more time in the corners (Figure 5P; *p* = 0.001), and less time in the center of the open field (Figure 5N; *p* = 0.021) as compared to wild-type mice. At this age, no differences were observed between the genotypes in the time spent sitting (Figure 5D; *p* = 0.349), rearing (Figure 5F; *p* = 0.359) and grooming (Figure 5L; *p* = 0.564).

Aging effects in the AβPP-PS1 mice were analyzed as relative values compared to the wild-type mice (set as 100%) of the corresponding age. With age, the time that AβPP-PS1 mice spent walking decreased, from 105.7%±3.8 at 8 months of age to 79.5%±7.1 at 12 months of age (*p* = 0.016), indicating decreased activity as disease progresses or decreased restlessness due to habituation to the open field. However, the traveled distance and mean velocity did not change with age (*p* = 0.105) as compared to age-matched wild-type animals (data not shown). Over time, AβPP-PS1 mice also spent more time in the corners of the open field (*p* = 0.018). Time spent in the corners of the open field increased from 115.7%±7.1 at 8 months of age to 169.3%±15.1 at 12 months of age, indicating increased anxiety-related behavior as disease progresses. The time spent sitting (*p* = 0.786), rearing (*p* = 0.481), grooming (*p* = 0.086), wall leaning (*p* = 0.087) and the time spent in the center of the open field (*p* = 0.116) did not change between 8 and 12 months of age (data not shown).

Altogether, these data show increased activity but decreased active exploration (rearing) in 8-month-old AβPP-PS1 mice compared to wild-type mice. This increased activity and decreased rearing disappear at 12 months of age, due to habituation to the open field and/or the older age of the animals. Instead, 12-month-old AβPP-PS1 mice show increased anxiety-related behavior compared to wild-type mice, as indicated by the increased time spent in the corners and decreased time spent in the center of the open field, and increased time spent wall leaning, which is a type of exploration but with an anxiety-related behavioral component [38], [50].

#### Morris water maze (MWM)

**Figure 6:**
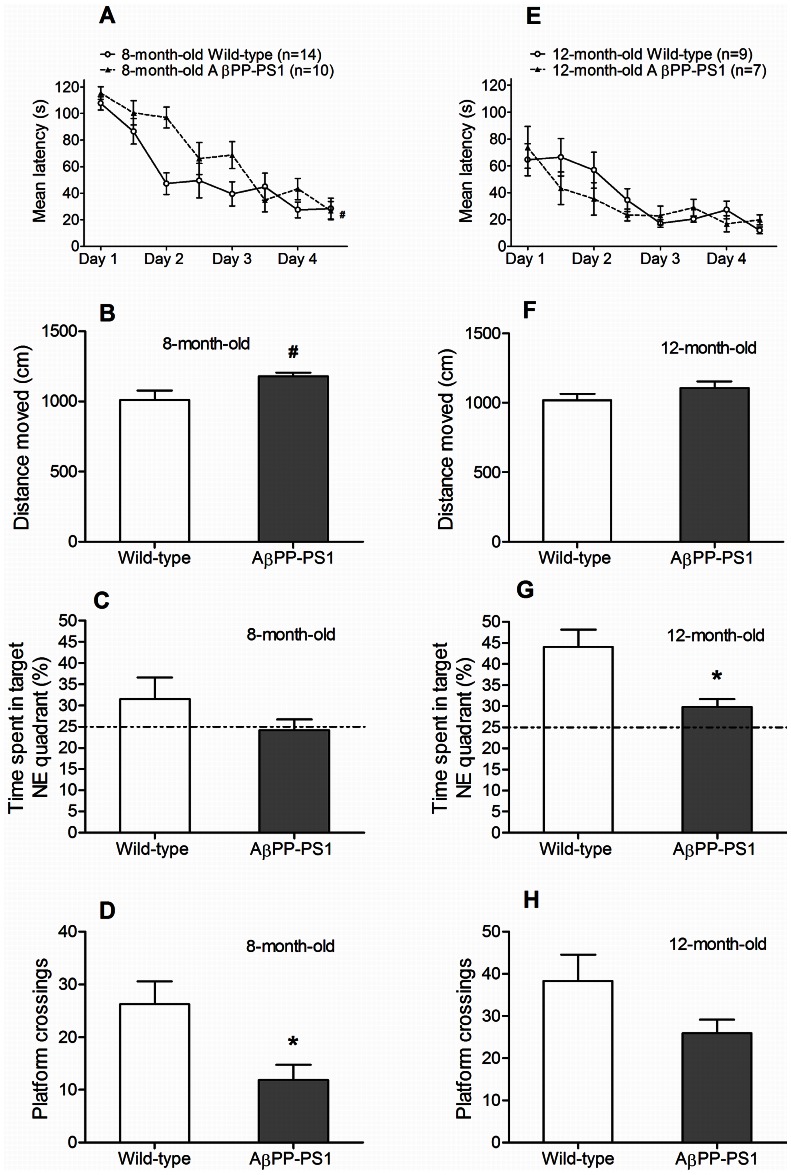
Morris water maze learning and memory in 8- and 12-month-old wild-type and AβPP-PS1 mice. Spatial learning was measured in a 4-day acquisition phase, by determining the latency to find a hidden platform in the NE quadrant. Spatial memory was tested in the probe phase in which the percentage of time spent in the target NE quadrant was measured and the total number of platform crossings (where formerly the platform had been located). A: Both 8-month-old wild-type (n = 14) and AβPP-PS1 mice (n = 10) showed a decrease in latency during training. Overall latencies tended to be higher in AβPP-PS1 mice, although it did not reach statistical significance, # trend *p* = 0.060. B: During the probe trial, the 8-month-old AβPP-PS1 mice traveled a slightly longer distance than wild-type animals, although it did not reach statistical significance, # trend *p* = 0.056. C: No differences were observed between the 8-month-old mice in the percentage of time spent in the target NE quadrant, although only wild-type mice deviated from 25% chance performance level. D: 8-month-old AβPP-PS1 mice crossed the exact platform location less often than wild-type mice, *p<0.05. E: Both 12-month-old wild-type (n = 9) and AβPP-PS1 mice (n = 7) showed a decrease in latency during training. Overall latencies did not differ between the genotypes. F: During the probe trial, no differences were observed in the distance moved between 12-month-old wild-type and AβPP-PS1 mice. G: 12-month-old AβPP-PS1 mice spent less time in the target NE quadrant, although both groups performed above 25% chance level, *p<0.05. H: No differences were observed between the 12-month-old mice in the frequency of platform crossings.

Both AβPP-PS1and wild-type mice showed a decrease in escape latency during training at 8 months of age (Figure 6A; *p*<0.001). However, wild-type mice learned to find the location of the hidden platform faster than the AβPP-PS1 mice, indicated by a significant time*genotype interaction (*p* = 0.009). Overall escape latencies tended to differ between wild-type and AβPP-PS1 (*p* = 0.060) at 8 months of age, although it did not reach statistical significance. Especially on day 2 of the acquisition phase, AβPP-PS1 mice showed higher escape latencies than wild-type mice, suggesting that spatial learning might be mildly affected at this age.

During the probe trial at 8 months of age, no differences were found between wild-type and AβPP-PS1 mice in the time spent in the platform quadrant (NE) (Figure 6C; *p* = 0.266). However, only wild-type mice deviated from 25% chance performance level, suggesting that only wild-type mice showed good memorization of the platform quadrant. Furthermore, AβPP-PS1 mice crossed the exact platform location less often than wild-type mice (Figure 6D; *p* = 0.019), reflecting impaired spatial memory for the exact platform location. No significant differences were found between the genotypes in the total distance moved (Figure 6B; *p* = 0.056) and mean swim velocity (*p* = 0.078; wild-type 17.5±1.2 cm/s, AβPP-PS1 20.4±0.5 cm/s) during the probe trial, although the AβPP-PS1 mice tended to be slightly more active than the wild-type animals.

Both wild-type and AβPP-PS1 animals learned to find the platform during acquisition at 12 months of age (Figure 6E), as indicated by a decrease in latency over trials (*p*<0.001). Escape latencies did not differ between the wild-type and AβPP-PS1 mice (*p* = 0.525), indicating that spatial learning was no longer affected by genotype at this age, most likely due to the repetition of this task.

AβPP-PS1 mice spent less time in the NE target quadrant compared to their wild-type littermates at 12 months of age (Figure 6G; *p* = 0.013), although both groups performed above 25% chance level, indicating memorization of the platform quadrant. No differences were found between the genotypes in the frequency of platform crossings (Figure 6H; *p* = 0.131), the total distance moved (Figure 6F; *p* = 0.221) and mean swim velocity (*p* = 0.232; wild-type 17.2±0.8 cm/s, AβPP-PS1 18.7±0.8 cm/s) during the probe trial.

Aging effects in the AβPP-PS1 mice were analyzed as relative values compared to the wild-type mice (set as 100%) of the corresponding age. Escape latencies during training in the MWM decreased with age (*p* = 0.001), such that the average escape latency of all 4 days was 128.2%±6.5 at 8 months of age relative to age-matched wild-type animals and 88.2%±7.2 at 12 months of age, indicating that 12-month-old AβPP-PS1 mice found the location of the hidden platform faster than 8-month-old AβPP-PS1 mice, most likely due to habituation to and repetition of the MWM task at 12 months of age. The time spent in target NE quadrant (p = 0.380), the number of platform crossings (p = 0.167), the distance moved (*p* = 0.121), and mean swim velocity (*p* = 0.173) did not change between 8 and 12 months of age (data not shown).

#### Reverse Morris water maze (rMWM)

**Figure 7:**
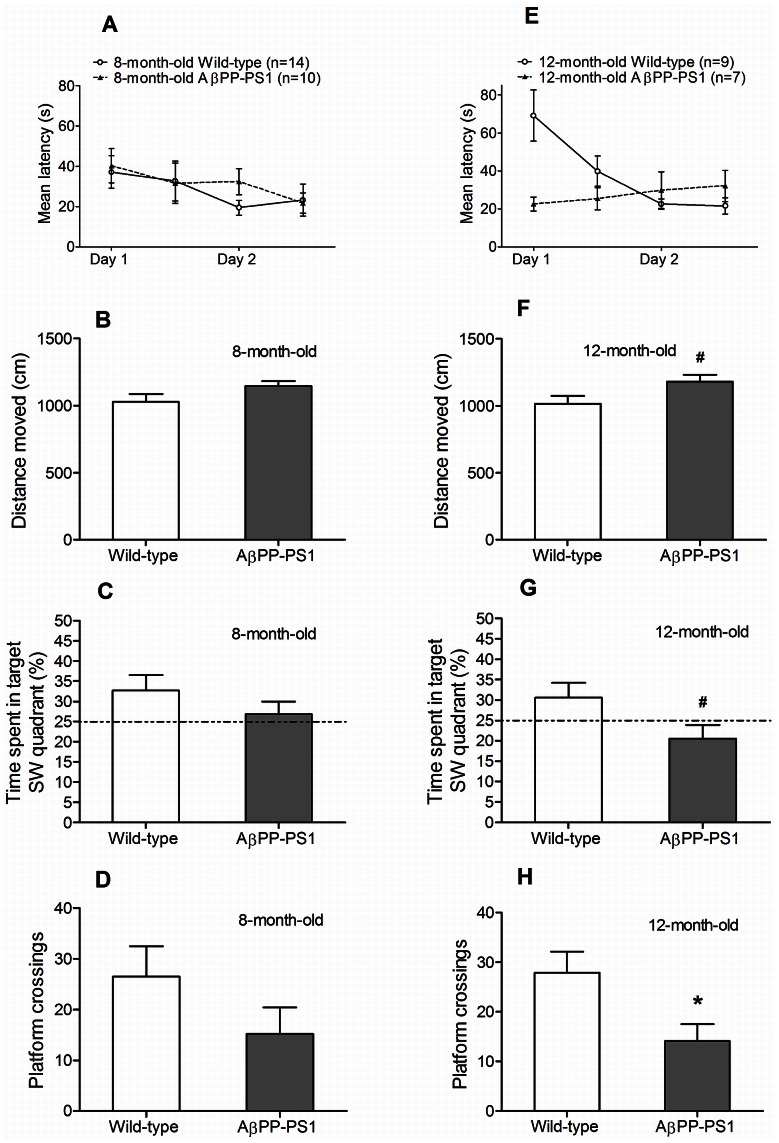
Reverse Morris water maze learning and memory in 8- and 12-month-old wild-type and AβPP-PS1 mice. Spatial learning with an extra episodic memory component was measured in a 2-day acquisition phase, by determining the latency to find a hidden platform in the SW quadrant. Spatial memory was tested in the probe phase in which the percentage of time spent in the target SW quadrant was measured and the total number of platform crossings (where formerly the platform had been located). A: Both 8-month-old wild-type (n = 14) and AβPP-PS1 mice (n = 10) showed a decrease in latency during training. Overall latencies did not differ between the genotypes. B: During the probe trial, the 8-month-old wild-type and AβPP-PS1 mice traveled a similar distance. C: No differences were observed between the 8-month-old mice in the percentage of time spent in the target SW quadrant, although only wild-type mice deviated from 25% chance performance level. D: No differences were observed between the 8-month-old mice in the frequency of platform crossings E: Only 12-month-old wild-type mice (n = 9) showed a decrease in latency during training. 12-month-old AβPP-PS1 mice (n = 7) did not improve their performance during acquisition. However, overall latencies did not differ between the genotypes. F: During the probe trial, the 12-month-old AβPP-PS1 mice traveled a slightly longer distance than wild-type animals, although it did not reach statistical significance, # trend *p* = 0.066. G: 12-month-old AβPP-PS1 mice tended to spent less time in the target SW quadrant, although it did not reach statistical significance, # trend *p* = 0.066. Only wild-type animals deviated from 25% chance performance level. H: 12-month-old AβPP-PS1 mice crossed the exact former platform location less often than wild-type animals, *p<0.05.

Both wild-type and AβPP-PS1 animals learned to find the new SW platform location during acquisition at 8 months of age (Figure 7A), as indicated by a decrease in latency over trials (*p* = 0.046). Overall escape latencies did not differ between wild-type and AβPP-PS1 (*p* = 0.693).

No differences were found between wild-type and AβPP-PS1 in the time spent in the SW quadrant during the probe trial at 8 months of age (Figure 7C; *p* = 0.271). However, only wild-type mice deviated from 25% chance performance level, suggesting that only wild-type mice showed good memorization of the new platform quadrant. No differences were found between the genotypes in the number of platform crossings (Figure 7D; *p* = 0.187), total distance moved (Figure 7B; *p* = 0.129) and mean swim velocity (*p* = 0.111; wild-type 17.6±1.0 cm/s, AβPP-PS1 19.8±0.7 cm/s) during the probe trial.

At 12 months of age, the wild-type animals showed a significant decrease in escape latency over time in the acquisition phase (Figure 7E; *p* = 0.001), indicating spatial learning. In contrast, escape latencies did not change over time in the AβPP-PS1 mice at 12 months of age (*p* = 0.649), due to unexpected low latencies at the initial trials. In spite of that, there was no difference in average escape latencies between AβPP-PS1 and wild-type animals (*p* = 0.134).

During the probe trial at 12 months of age, AβPP-PS1 mice tended to spent less time in the target SW quadrant than wild-type mice (Figure 7G; *p* = 0.066), although it did not reach statistical significance. Furthermore, only wild-type mice deviated from 25% chance performance level, suggesting that only wild-type mice showed good memorization of the new platform quadrant. AβPP-PS1 mice also crossed the former platform location significantly less often than wild-type animals (Figure 7H; *p* = 0.029), reflecting impaired spatial memory as well. No significant differences were found between the genotypes in the total distance moved (Figure 7F; *p* = 0.066) and mean swim velocity (*p* = 0.066; wild-type 17.1±1.0 cm/s, AβPP-PS1 19.9±0.9 cm/s), although the AβPP-PS1 mice tended to be slightly more active than the wild-type animals.

Aging effects in the AβPP-PS1 mice were analyzed as relative values compared to the wild-type mice (set as 100%) of the corresponding age. Escape latencies during training in the rMWM did not change with age and repetition of the rMWM test (*p* = 0.123).

During the probe trial, the swim distance increased with age (*p* = 0.049) from 111.4%±3.7 at 8 months to 116.2%±5.2 at 12 months, indicating hyperactivity as disease progresses. The time spent in the SW target quadrant (*p* = 0.165) and number of platform crossings (*p* = 0.456) did not change between 8 and 12 months of age (data not shown).

### Magnetic Resonance Spectroscopy (MRS)

**Figure 8:**
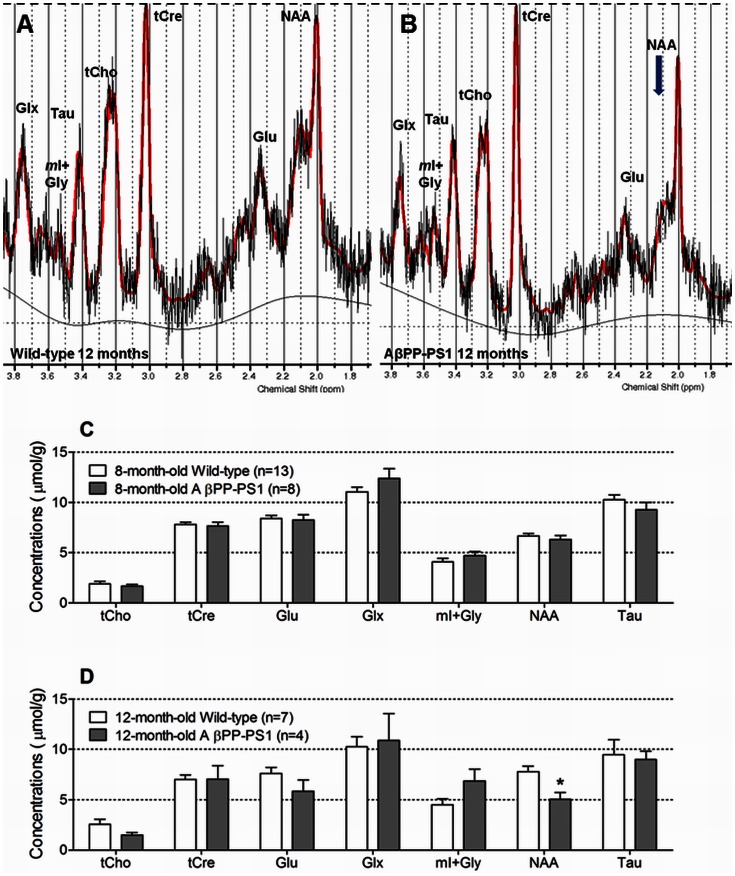
Neurochemical profile of the hippocampus measured by single voxel ^1^H MRS at 7 Tesla. A: Representative ^1^H MR spectra acquired from the hippocampus of a 12-month-old wild-type mouse. B: Representative ^1^H MR spectra acquired from the hippocampus of a 12-month-old AβPPswe-PS1dE9 transgenic mouse. Notice the decreased NAA peak in AβPP-PS1compared to wild-type. C: At 8 months of age, no differences between wild-type (n = 13) and AβPP-PS1 mice (n = 8) were observed in the hippocampal neurochemical profile. D: At 12 months of age, AβPP-PS1 mice (n = 4) had significantly lower concentrations of NAA than wild-type mice (n = 7), *p<0.05. tCho = choline-containing compounds; tCre = creatine and phosphocreatine; Glu = glutamate; Glx = glutamine and glutamate; *m*I+Gly = *myo*-Inositol and glycine; NAA = *N*-acetylaspartate; Tau = taurine.

No differences were observed in the neurochemical profile of the hippocampus of 8-month-old wild-type and AβPP-PS1 mice (Figure 8C). Wild-type and AβPP-PS1 mice had similar concentrations of choline-containing compounds (tCho; *p* = 0.514), creatine and phosphocreatine (tCre; *p* = 0.712), glutamate (Glu; *p* = 0.785), glutamine and glutamate (Glx; *p* = 0.181), *myo*-Inositol and glycine (*m*I+Gly; *p* = 0.272), *N*-acetylaspartate (NAA; *p* = 0.456), and taurine (Tau; *p* = 0.245).

At 12 months of age, AβPP-PS1 mice had significantly lower concentrations of NAA (*p* = 0.012) than wild-type mice, indicating decreased neuronal health and/or increased neurodegeneration (Figure 8D). No differences were observed between wild-type and AβPP-PS1 mice in the levels of tCho (*p* = 0.123), tCre (*p* = 0.986), Glu (*p* = 0.162), Glx (*p* = 0.807), *m*I+Gly (*p* = 0.082) and Tau (*p* = 0.804).

Aging effects in the AβPP-PS1 mice were analyzed as relative values compared to the wild-type mice (set as 100%) of the corresponding age. With age, the concentration of NAA decreased from 94.7%±6.0 at 8 months of age to 65.1%±8.4 at 12 months of age (*p* = 0.017), indicating decreased hippocampal neuronal viability as disease progresses.

### Immunohistochemistry

**Figure 9:**
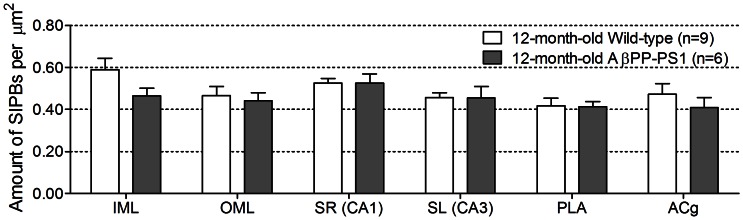
Amount of synaptophysin-immunoreactive presynaptic boutons (SIPBs) per µm^2^ in 12-month-old wild-type and AβPP-PS1 mice. The amount of SIPBs were quantified in the hippocampal inner (IML) and outer molecular layer (OML) of the dentate gyrus, the stratum radiatum (SR) of the CA1 area, and the stratum lucidum (SL) of the CA3 area, and in the cortical prelimbic area (PLA) and anterior cingulate gyrus (ACg). No differences in the amount of SIPs between 12-month-old wild-type (n = 9) and AβPP-PS1 mice (n = 6) were observed in any region analyzed (*p*>0.10).

At 12 months of age, there were no significant differences in the amount of synaptophysin-immunoreactive presynaptic boutons (SIPBs) between AβPP-PS1 and wild-type mice (Figure 9) in any of the regions analyzed (*p*>0.10). Furthermore, no significant differences were found in the amount of doublecortin-positive (Dcx+) immature neurons between 12-month-old AβPP-PS1 and wild-type mice (*p* = 0.082; wild-type 9.2±1.5 Dcx+ cells, n = 6; AβPP-PS1 5.5±0.3 Dcx+ cells, n = 4).

### Amyloid-β Measures

Correlation analyses with the extracellular amyloid-β deposition, TBS-T soluble Aβ levels and high-molecular weight Aβ aggregate levels found in the brains of 12-month-old AβPP-PS1 mice [34] were performed using bivariate Pearson’s correlation method.

No significant interactions were found between any of the Aβ measures and the open field data (*p*>0.05), and the MWM and rMWM acquisition and probe data (*p*>0.05).

A significant negative correlation was found between the Aβ plaque load in the hippocampus and the tCho levels measured with ^1^H MRS (*p* = 0.005; R = −0.995). Furthermore, increased *m*I+Gly levels significantly correlated with increased levels of the high-molecular weight Aβ_40_ (*p* = 0.007; R = 0.993) and Aβ_42_ (*p* = 0.003; R = 0.997) aggregates.

A significant negative correlation was found between the TBS-T soluble Aβ_42_ levels and the amount of SIPBs in the PLA region (*p* = 0.040; R = −0.895). In contrast, positive correlations were found between the amount of SIPBs in the SR region of the hippocampus and the levels of high-molecular weight Aβ_40_ (*p* = 0.009; R = 0.962) and Aβ_42_ (*p* = 0.027; R = 0.920) aggregates. However, these correlations between the amount of SIPBs and Aβ measures did no longer reach significance when Bonferroni’s correction for multiple comparisons was applied (*p*>0.008).

Finally, we found a significant negative correlation between the amount of immature neurons and the level of high-molecular weight Aβ_40_ (*p* = 0.041; R = −0.959) aggregates, suggesting impaired neurogenesis with increasing levels of Aβ_40_ aggregates.

## Discussion

The present longitudinal study set out to characterize the neurochemical profile of the hippocampus, measured by ^1^H MRS, in the brains of AβPPSswe-PS1dE9 mice at 8 and 12 months of age as compared to age-matched wild-type littermates. Furthermore, we wanted to determine whether alterations in hippocampal metabolite levels coincided with behavioral changes, cognitive decline and neuropathological features, to gain a better understanding of the underlying neurodegenerative processes. Moreover, we determined the extracellular amyloid-β load, TBS-T soluble Aβ levels and high-molecular weight Aβ aggregate levels of the 12-month-old AβPP-PS1 mice in our laboratory [34]. We performed correlation analyses using bivariate Pearson’s correlation method to gain a better understanding of the possible involvement of Aβ in neurochemical and behavioral changes, cognitive decline and neuropathological features in AβPP-PS1 transgenic mice.

In agreement with previous results from our lab [38], 8-month-old AβPP-PS1 mice already display behavioral changes in the open field, such as increased locomotor activity and decreased exploration. The increased activity is a specific characteristic of many AβPP transgenic mice [51]–[54] and may be explained as a result of elevated anxiety levels [53], [55] or impaired habituation learning [51], [52]. In our view increased anxiety is most likely, since the AβPP-PS1 mice also showed less rearing behavior (exploring the environment) compared to age-matched wild-type mice. Curiosity motivates mice to explore a novel environment, but this exploratory drive is in conflict with fear of the unknown. Moreover, 12-month-old AβPP-PS1 mice also showed increased anxiety-related behavior, as indicated by the increased time spent in the corners of the open field, increased time spent wall leaning, and decreased time spent in the center of the open field (i.e. anxious mice prefer the borders of the open field). Increased anxiety and restlessness, as noticed as hyperactivity in several AβPP transgenic mouse models, also occur in AD patients [56], [57]. However, activity levels decreased between 8 and 12 months of age both in AβPP-PS1 and wild-type mice, suggesting increased familiarity with the open field even after 4 months.

In line with previous results from our lab, AβPP-PS1 mice also showed impaired performance in the MWM and rMWM both at 8 and 12 months of age as compared to wild-type littermates [38], which might suggest visuo-spatial learning and memory deficits. Noteworthy are the relatively low escape latencies of both AβPP-PS1 and wild-type mice during the initial trials of the rMWM training at 8 months. Although 8-month-old wild-type mice displayed good short-term spatial memory for the NE platform location during the probe of the MWM, it appears that this information might not have been sufficiently consolidated in the long-term memory since they displayed more “random” search during the initial rMWM trials (resulting in lower escape latencies) than expected if animals had good long-term memory for the former platform location. One possible explanation could be that the MWM task as used in the current study was slightly too difficult to optimally master within 4 days of training. For mice, a MWM pool with a diameter of 120 cm and a platform of 10−12 cm in diameter (or even larger) is commonly used, resulting in a search area to target size of 144∶1 to 100∶1 (or even lower). In the current study, we used a pool with a diameter of 104 cm and a platform of 8 cm in diameter, resulting in a search area to target size of 169∶1, thereby increasing the MWM task difficulty [58], [59]. At 12 months of age, when the mice were exposed to the MWM for the second time, wild-type mice not only showed a higher preference for the NE target quadrant during the probe than at 8 months of age, they also persevered in searching the original platform location during the initial trials in the rMWM, suggesting that habituation to, and repetition of the MWM task resulted in enhanced short-term, and formation of long-term memory for the platform location in wild-type mice.

Standard measures of performance in the MWM as used in the current study, such as escape latency during acquisition training, time spent in platform quadrant and the number of platform crossings during a probe trial, may depend on other factors than visuo-spatial learning ability and memory capacity alone. Longer escape latencies during spatial navigation may be caused by slower swim speed, although we do not expect this to be a confounding factor, since wild-type and AβPP-PS1 mice did not display any significant differences in swim speed during the probe trials at any age. Furthermore, several groups have reported no differences in swim speed during MWM training between C57BL6/J wild-type and AβPPswe-PS1dE9 mice at any age tested [60]–[63]. Longer escape latencies during spatial navigation may also be caused by the use of certain, less efficient, search strategies, such as a constant random search of the entire surface area of the pool, which would indicate a complete lack of spatial learning abilities, or by persistent performance of a less efficient than spatial (direct) search strategy, such as circling the pool at a certain distance from the wall to find the platform [64]–[67]. In this strategy, mice have not used the spatial cues to learn the location of the platform, although such a strategy would result in a successful location of the escape platform during training. To our knowledge, the search strategies used by AβPP-PS1 mice in the MWM have not be determined yet and should be addressed in future studies to further characterize the cognitive deficits in the AβPPswe-PS1dE9 mouse model. However, our results might imply that the AβPP-PS1 mice made use of a random or persistent non-spatial search strategy to locate the platform during acquisition training, since AβPP-PS1 mice showed slightly longer escape latencies to find the hidden platform in the MWM at 8 months of age, searched the target quadrant during the probe trials at chance level (∼25% or less) and they crossed the former platform location less often than wild-type mice. In contrast, wild-type mice performed well above chance level during the probe trials, indicating memorization of the platform location, which might imply that they made use of a more spatially precise search strategy. Furthermore, the high latency of the wild-type mice during the first 2 acquisition trials of the reversal task at 12 months of age might indicate that they learned and remembered the original NE platform location quite well, and persevered in searching the original platform location during those initial trials in the reversal MWM. The 12-month-old AβPP-PS1 mice in contrast did not improve their performance over time in the reversal task, but seemed to perform better in the first 2 acquisition trials compared to their wild-type littermates. Again this might imply that the AβPP-PS1 mice made use of a more random non-spatial search strategy and thereby coincidentally reaching the platform faster than their wild-type littermates.

This is in line with a study by O’Leary and Brown, in which the search strategies used by 16-month-old AβPP-PS1and wild-type mice during visuo-spatial navigation in the Barnes Maze were analyzed [68]. 16-month-old AβPP-PS1 mice predominantly made use of a random search strategy to locate the escape hole in the Barnes Maze, whereas wild-type mice predominantly made use of a spatial (direct and accurate) search strategy. Similar results have been found for the search strategies used by the TgCRND8 transgenic AβPP mouse model in the MWM test [64], [65]. Moreover, it was shown that C57BL6 mice treated with Temozolomide (TMZ) to suppress adult hippocampal neurogenesis, displayed a delayed (or even absent) use of directed and place specific search patterns in the (reversal) MWM test compared to untreated mice, suggesting that hippocampal neurogenesis is necessary for adding flexibility to some hippocampus-dependent qualitative parameters of learning [67]. Although we did not observe a significant decrease in the amount of immature neurons in the 12-month-old AβPP-PS1 mice in our current study, reduced hippocampal neurogenesis has been found previously in AβPP-PS1 mice [69]–[71] and in AD patients [72], [73], and might underlie some aspects of the cognitive deficits in AD.

Synaptic loss is a pathological hallmark of AD, and it is the best correlate of cognitive impairment [2], [74]–[76]. Synaptophysin is a widely used marker for quantification of presynaptic terminals, but conflicting results have been reported on synaptophysin immunoreactivity (SYN-IR) both in AD patients [77]–[80] and AD mouse models. SYN-IR in transgenic mice that develop Aβ plaques has been reported to decline, increase, or not change, apparently depending on the interplay of mouse strain, transgene, age, and disease progression [49], [81]–[86]. In the current study, no changes were found in the amount of presynaptic boutons at 12 months of age, similar to earlier reports of no changes in the amount of presynaptic boutons in 8-month-old AβPP-PS1 mice [42], but in contrast to the increased amount of presynaptic boutons found in the hippocampus of 15-month-old AβPP-PS1 mice, which was suggested to reflect a synaptic compensatory response to maintain connectivity and preserve cognitive functioning [42]. However, we did find positive correlations between the levels of high-molecular weight Aβ_40_ and Aβ_42_ aggregates and the amount of SIPBs in the SR region of the hippocampus in the 12-month-old AβPP-PS1 mice, which might suggest a possible involvement of Aβ aggregates in the initiation of this synaptic compensatory response. In contrast, a negative correlation was found between the TBS-T soluble Aβ_42_ levels and the amount of SIPBs in the PLA region, which might suggest a possible involvement of soluble Aβ oligomers in synaptic loss. However, these correlations between the amount of SIPBs and Aβ measures did no longer reach significance when Bonferroni’s correction for multiple comparisons was applied. Since the necessity of using the conservative Bonferroni’s adjustment for multiple correlations is still debated [87]–[89], these findings must be interpreted with caution.

Recent studies suggest that synapse loss might not be an early event in the progression of AD, as a decrease in synapses is only seen in later stages of the disease, where especially tau pathology is more widespread [90]–[92]. It has been proposed that the pathogenesis of synaptic damage in AD can be divided into two phases: in the first phase, the disease is characterized by neuronal and synaptic dysfunction (loss of plasticity), which triggers a compensatory response to maintain connectivity by, first the formation of new synapses, and later on by increasing the size of remaining synapses [75], [86], [90], [93]. In the second phase, cycles of aberrant sprouting and neuritic disorganization eventually result in synapse loss and neurodegeneration [94], [95]. It could be hypothesized that the decreased hippocampal neuronal viability found in our 12-month-old AβPP-PS1 mice, together with increasing levels of Aβ_40_ and Aβ_42_ oligomers and aggregates, contribute to synaptic and neuronal dysfunction, which might trigger a synaptic compensatory response that in time results in the increased amount of presynaptic terminals as seen in the hippocampus of 15-month-old AβPP-PS1 mice [42].

*In vivo* ^1^H MRS has been used to characterize cerebral metabolic alterations in mild cognitive impaired (MCI) individuals and AD patients [23]–[25], [96]–[99], and in several transgenic AβPP animal models for AD [33], [100]–[107]. The most consistent finding is a decrease in NAA level, a biomarker for neuronal integrity, in the cortex and hippocampus with aging and disease progression. Clinically, reduction of NAA has been used as an indicator of the progression of neurodegenerative pathology in AD patients, and to differentiate stable MCI from progressive MCI [23], [96], [97], [108], [109]. Longitudinal changes in ^1^H MRS measures have also been evaluated in AD patients during therapeutic trials with cholinergic agents. Treatment with xanomeline, an M1-selective cholinergic agonist, increased the level of NAA and decreased the level of Cho compared to baseline values before treatment. Changes in Cho levels from baseline further correlated with improved or at least stable ADAS-Cog scores [110], [111]. These findings not only support the feasibility of MRS measures in AD clinical trials, but also indicate that AD-related changes detected by MRS may be reversible, and may reflect aspects of neuronal integrity or function. Furthermore, many previous ^1^H MRS studies have found increased levels of *m*I in the temporal, parietal and occipital lobes of AD patients [22], [25], [98], [99]. *m*I is a sugar alcohol that is thought to be a marker for osmotic stress, astrogliosis and microglial activation [27], and an increase in cerebral *m*I levels is therefore associated with inflammatory processes. Although we did not observe significant differences in hippocampal *m*I+Gly concentration between the 12-month-old wild-type and AβPP-PS1 mice in the current study, correlation analyses revealed a positive correlation between the levels of Aβ_40_ and Aβ_42_ aggregates and *m*I+Gly levels in the AβPP-PS1 animals, suggesting a possible involvement of Aβ in inflammatory processes.

In transgenic animals models, significant reduction of NAA levels and elevation of *m*I levels were found to occur at different ages in different transgenic species apparently depending on the interplay of mouse strain, transgene and disease progression. The AβPPswe (Tg2576) model and the AβPPswe-PS2N1411 (PS2APP) model display significantly decreased NAA level in the frontal cortex at 19–24 months of age, when Aβ deposits are widespread, but on the other hand no change in *m*I levels compared to age-matched wild-type mice [100], [102]. In the case of the AβPPswe-PS1M146L model, a significant reduction in the NAA/tCre ratio in the frontal hippocampus was observed at 16 month of age in one study [101], but already at 6.5 months in another study [104], while the most profound increase in *m*I level was observed after 20 months of age [101], [103], [104]. In contrast to our findings of a lower NAA concentration in the hippocampus of 12-month-old AβPPswe-PS1dE9 mice, Xu and colleagues did not observe significantly decreased hippocampal NAA/total creatine (tCre) levels until 16 months of age, which was associated with progressive degeneration of CA3 pyramidal neurons [107]. Chen and colleagues on the other hand observed a significantly lower concentration of NAA in the frontal cortex and hippocampus already at 5 months of age in the AβPP-PS1mouse model, which coincided with neuronal loss and neuronal shrinkage. Furthermore, compared to age-matched wild-type animals the concentration of *m*I was significantly higher in 3-month-old AβPP-PS1 mice, and pathology showed activation and proliferation of astrocytes in the frontal cortex and hippocampus [112], [113]. The discrepancy between the results found in the latter two studies and our findings at 8 and 12 months of age are most likely due to differences in methodology, e.g. the field strength of the MR system, the acquisition parameters used, the exact position of the spectroscopic volume of interest, and the amount of animals measured.

It has been demonstrated that AβPPswe-PS1dE9 mice may exhibit various neurobiological abnormalities in the hippocampus before 8 months of age. Such abnormalities include inflammatory processes involving clusters of activated microglia and astrocytes, and TNF-α expression [114], functional pre- and postsynaptic cholinergic deficits [115], impaired survival of newborn neuronal cells [116], and increasing levels of insoluble and soluble Aβ40 and Aβ42 in parenchyma and vessel walls [36], [53], [117]. These neurobiological alterations might underlie the mild behavioral changes and cognitive decline observed in our 8-month-old AβPP-PS1 mice in the open field and (r)MWM, since cholinergic neurotransmission and the integration of newborn neuronal cells into the circuitry of the hippocampus are important for learning and memory [67], [118], [119]. Although we did not observe a decrease in the amount of immature neurons in our 12-month-old AβPP-PS1mice, reduced hippocampal neurogenesis has been found previously in AβPP-PS1 mice beyond 8 months of age [69]–[71], as well as expression of IL-1β, IL-6 and MCP-1, suggesting chronic inflammatory processes [114], decreased cerebral blood volume (CBV) [34] and gray and white matter degeneration [120]. Increasing levels of Aβ together with chronic inflammation and decreased delivery of oxygen and nutrients (due to cerebral hypoperfusion) can cause neuronal dysfunction and suppress hippocampal neurogenesis [17], [19], [121], thereby contributing to the more severe cognitive and behavioral dysfunction observed in our 12-month-old AβPP-PS1 mice alongside with a reduction in NAA levels measured with ^1^H MRS. Furthermore, beyond 12 months of age AβPP-PS1 mice display more progressive AD-like pathology including increased brain-derived neurotrophic factor (BDNF) levels and lower norepinephrine, serotonin and acetylcholine levels [122], [123], hippocampal atrophy and a reduced amount of glucose-transporter type-1 [124], increased amount of presynaptic boutons [42], and a decrease in CBV [125]. However, it should be noted that AβPP-PS1 mice do not develop the widespread neurofibrillary tangle pathology or extensive neurodegeneration as seen in AD patients. Thus it is important to use caution in interpreting results found in AβPP-PS1 (and other transgenic) mice and translating them to the human AD situation.

To summarize, in this paper we characterized the neurochemical profile of the hippocampus, measured by ^1^H MRS at 7 Tesla, in the brains of 8- and 12-month-old AβPPswe-PS1dE9 mice as compared to age-matched wild-type animals. Our results show that at 8 months of age no alterations in hippocampal metabolite levels could be detected, while behavioral changes and cognitive decline were present in the AβPP-PS1 mouse model. At 12 months of age, a decrease in hippocampal NAA levels, reflecting reduced neuronal integrity, correlated with more severe behavioral and cognitive impairment in AβPP-PS1 mice as compared to wild-type animals. Furthermore, correlation analyses suggest a possible role of Aβ in inflammatory processes, synaptic dysfunction and impaired neurogenesis.

^1^H MRS could potentially provide unique information about the underlying degenerative processes, because metabolite levels are sensitive to different *in vivo* pathological processes at the molecular or cellular level. Furthermore, ^1^H MRS has great potential for the early diagnosis of AD, monitoring disease progression and evaluating the efficacy of potential therapeutic agents, both in animals models and AD patients. However, to observe small changes related to the disease progression, ^1^H MRS data need to be acquired from a large number of mice. Furthermore, the sensitivity and specificity of ^1^H MRS depend on the field strength of the MR system, the acquisition parameters and the size and position of the spectroscopic volume of interest. Therefore, we cannot exclude the presence of subtle metabolic alterations at 8 months of age, since our ^1^H MRS methodology may not have been sensitive enough to detect small but important functional or anatomical abnormalities in the AβPP-PS1mouse hippocampus.
